# A Proposal for Multidisciplinary Tele-Rehabilitation in the Assessment and Rehabilitation of COVID-19 Survivors

**DOI:** 10.3390/ijerph17134890

**Published:** 2020-07-07

**Authors:** Abayomi Salawu, Angela Green, Michael G. Crooks, Nina Brixey, Denise H. Ross, Manoj Sivan

**Affiliations:** 1 Hull University Teaching Hospital National Health Service (NHS) Trust, Hull HU16 5JQ, UK; angela.green@hey.nhs.uk (A.G.); michael.crooks@hey.nhs.uk (M.G.C.); nina.brixey@hey.nhs.uk (N.B.); 2 Hull York Medical School, University of Hull, Hull HU6 7RX, UK; 3 Department of Sport, Health and Exercise Science, University of Hull, Hull HU6 7RX, UK; 4 Leeds Teaching Hospitals NHS Trust, Leeds LS9 7TF, UK; Denise.Ross@nhs.net (D.H.R.); M.Sivan@leeds.ac.uk (M.S.); 5 Academic Department of Rehabilitation Medicine, University of Leeds, Leeds LS1 3EX, UK

**Keywords:** COVID-19, multi-disciplinary tele-rehabilitation, pulmonary rehabilitation

## Abstract

A global pandemic of a new highly contagious disease called COVID-19 resulting from coronavirus (severe acute respiratory syndrome (SARS)-Cov-2) infection was declared in February 2020. Though primarily transmitted through the respiratory system, other organ systems in the body can be affected. Twenty percent of those affected require hospitalization with mechanical ventilation in severe cases. About half of the disease survivors have residual functional deficits that require multidisciplinary specialist rehabilitation. The workforce to deliver the required rehabilitation input is beyond the capacity of existing community services. Strict medical follow-up guidelines to monitor these patients mandate scheduled reviews within 12 weeks post discharge. Due to the restricted timeframe for these events to occur, existing care pathway are unlikely to be able to meet the demand. An innovative integrated post-discharge care pathway to facilitate follow up by acute medical teams (respiratory and intensive care) and a specialist multidisciplinary rehabilitation team is hereby proposed. Such a pathway will enable the monitoring and provision of comprehensive medical assessments and multidisciplinary rehabilitation. This paper proposes that a model of tele-rehabilitation is integrated within the pathway by using digital communication technology to offer quick remote assessment and efficient therapy delivery to these patients. Tele-rehabilitation offers a quick and effective option to respond to the specialist rehabilitation needs of COVID-19 survivors following hospital discharge.

## 1. Introduction

In December 2019, a new highly contagious respiratory illness was observed to have developed in the Wuhan province of China. Over the following weeks, a rapid spread across the globe was observed as a result of what is now known to be infection with the severe acute respiratory syndrome (SARS)-CoV-2 virus, also called the coronavirus. The illness caused by the virus was labelled coronavirus disease or COVID-19 by the World Health Organization (WHO) on 11 February 2020 [1]. The infection was labelled a pandemic by the WHO on the 11th of March 2020, and they warned of the alarming level of inaction by various governments across the world that could further exacerbate the pandemic [2]. The disease presents with a broad clinical spectrum, with a majority (81%) of cases having a mild presentation with either no symptoms or mild upper respiratory tract infection symptoms/a mild pneumonia. About 14% of cases have severe disease with dyspnoea, increased respiratory rate, hypoxia, and/or lung infiltrates within 24−48 h. A small but significant minority (5%) develop critical disease with respiratory failure, septic shock, and/or multiple organ dysfunction/failure [3,4]. The treatment is mainly symptomatic, and oxygen therapy represents the major treatment intervention for patients with severe infection. There is still no specific curative drug treatment recommended for the disease, and no vaccine is currently available. Mechanical ventilation may be necessary in cases of respiratory failure refractory to non-invasive forms of respiratory support, and hemodynamic support is essential for managing septic shock [3]. A case fatality rate of about 4% has been reported from China [5]. In the United Kingdom (UK), a 33% mortality rate has been reported for hospitalized COVID-19 patients [6].

Two Chinese nationals from the same family staying at a hotel in York were the first reported cases in the UK [7]. Subsequently, other UK patients with recent travel histories to affected regions tested positive to the virus. The first known case of a person infected within the UK was reported on the 29th of February [8]. The first case fatality from the infection in the UK occurred on March 5th in an elderly patient with underlying health conditions at the Royal Berkshire Hospital NHS Trust. Thus far in the UK, a total of 307,980 people have tested positive to the virus, and 43,230 people have sadly died as of 26 June 2020 [9].

It is difficult to ascertain if premorbid fitness and wellbeing have a direct impact on the clinical course of the disease. It has been observed that lifestyle-related diseases such as obesity, insulin resistance and diabetes are linked to an increased risk of hospitalization and mortality. This has prompted suggestions that high levels of cardiorespiratory fitness might be protective to developing a severe case of COVID-19 [10]. These lifestyle-related diseases have a common linkage of sedentary behavior, poor dietary habits, and a lack of physical exercise. They are also characterized by chronic low-grade inflammation that is readily ameliorated by the positive effect of moderate doses of exercise on some immune markers associated with these diseases. Elderly patients and those with underlying medical conditions appear to be the most vulnerable group at risk to COVID-19. Evidence also indicates a markedly higher mortality risk to COVID-19 among Black, Asian, and Minority Ethnic (BAME) groups. Similar adverse outcomes are seen for BAME patients in intensive care units and amongst medical staff and healthcare workers. The exact reasons for this increased risk and vulnerability from COVID-19 in BAME populations are not known [11].

The planned response by the health authorities to the pandemic largely focused on the prevention of infection by limiting spread and ensuring that the acute care services, including intensive care units (ICUs), were able to cope with the care of patients with the disease. There has been very minimal discussion on the possible need for specialist rehabilitation services as part of the planning of the national response to COVID-19. Understandably, the disease has had a massive impact on health care services. Routine clinical activities have been disrupted as part of the emergency preparation for the expected surge in hospital bed utilization at the peak of infection. The disruption of clinical activities has had a massive impact on specialist and community rehabilitation services. Many of the patients served by the specialist rehabilitation service fall within the vulnerable group, and some may require regular intervention procedures such as botulinum toxin injections to manage the residual deficits from their condition. Specialist rehabilitation services in England are classified on a three-tier structure, as defined by NHS England into Levels 1, 2, and 3 [12], which align, respectively, with the tertiary, secondary, and primary levels of healthcare services. All levels of rehabilitation service provision have been impacted upon by the disease burden. Many patients undergoing inpatient rehabilitation were hurriedly discharged from hospital as soon as medical stability was achieved for the continuation of their rehabilitation in the community. This was to create capacity within the acute services for an anticipated surge at the peak of pandemic. However, community specialist rehabilitation provision has been historically sparse across various parts of the UK, notably in our region (Yorkshire and Humberside), and there have been concerns that these community services would be quickly overwhelmed. Furthermore, the need to maintain guideline rules of social distancing and also for community-dwelling adults to only undertake essential travels has had significant impact on the services provided by the community therapy teams. The closures of gyms and leisure centers, as well as the temporary cessation of social prescribing networks and activities, have further limited access to other potential rehabilitation resources.

Patients who recover from the infection, especially those that required ICU input, are likely to have on-going rehabilitation needs. In view of the novel nature of this disease, very little is known about the long-term residual deficits and the rehabilitation needs of this group of patients. While it is anticipated and expected that pulmonary rehabilitation (PR) will predominate during the acute episode with the need to improve oxygenation and facilitate the drainage of pulmonary secretions, the rehabilitation requirements following discharge is less known. It is assumed that those managed in the ICU may develop residual deficits such as critical illness neuropathy/myopathy and deconditioning commonly seen as part of critical care episodes.

Evidence from previous coronavirus-related respiratory tract viral infection epidemics such as the SARS, which swept the world in 2002−2003, and middle east respiratory syndrome (MERS), which emerged in 2012, indicate the development of impairment of physical, mental, and social functioning brought about by respiratory compromise and deconditioning [13,14,15]. There is also evidence that these patients developed neurological complications such as myopathy, neuropathy, and generalized deconditioning, which is often associated with prolonged stay within the intensive care environment [16]. Patients also develop psychological and mood-related effects following severe illness [17]. In addition to all this is the pervasive anxiety that is apparent within the general population during this pandemic episode. The psychological impact of the pandemic extends beyond the patients; it also affects the clinicians, therapists, and carers whose families, friends, and colleagues might be affected by the disease. The risk of contracting the infection by frontline health care workers and carers is heightened by the long hours and stressful work environment prevalent during the pandemic. This is further exacerbated by the difficulty in accessing appropriate personal protective equipment (PPE) such as high-quality face masks, gowns, and gloves in community care settings. Accelerated hospital discharge procedures for medically stable patients with COVID-19 might have contributed to the disease burden in the care home sector [18]. Poor compliance with infection prevention and control measures may also have heightened the risk of providing care in the community [19].

This paper proposes a model of a care pathway to mitigate against the impact on the rehabilitation services due to the response of the UK National Health Service in managing the COVID-19 crisis. The value of performing rehabilitation assessment and intervention for COVID-19 survivors alongside the mandatory medical follow-up requirements is elaborated. The issues of capacity and alternative ways of working within the rehabilitation services are explored, and tele-rehabilitation is proposed as a viable alternative to traditional face-to-face intervention. This paper advocates for a tele-rehabilitation pathway model based on the case of need. The proposal was influenced by the clinical experience of the authors, the clinical exigencies of this novel disease, and the local/regional service peculiarities.

## 2. Rehabilitation Interventions in COVID-19

Rehabilitation is a key component of recovery following illnesses and major health interventions. It is an established clinical intervention in the management of patients with various clinical conditions and has been demonstrated to be effective in optimizing clinical outcomes. Most of the evidence for the effectiveness of rehabilitation has been derived from organized stroke care [20]. Factors that significantly influence rehabilitation outcomes include the timeliness of intervention, the intensity, and the frequency of input. Clinical evidence indicates that therapy (physical, occupational, psychological, speech and language) improve functional outcomes in patients with neurological conditions, especially stroke [20]. The improvement in outcome follows a dose–response relationship: the greater the therapy dose, the better the functional outcome [21]. Many patients, however, receive suboptimal doses of therapy due to resource limitation and access issues. This is more likely to be exacerbated in the current prevailing climate of the COVID-19 pandemic. As more patients with COVID-19 survive the acute illness episodes, the problem of unmet rehabilitation needs is likely to increase with time.

Evidence published by Chinese rehabilitation specialists identified the need for PR for survivors of the coronavirus infection. Pulmonary rehabilitation is recommended to improve lung function, exercise tolerance, and reduce fatigue post-COVID-19, particularly for those who required hospitalization [22]. Pulmonary rehabilitation is already an established clinical management strategy for patients with chronic lung conditions. There are existing guidelines in the UK issued by the National Institute for Health and Care Excellence [23] with regards to the rehabilitation of patients following critical illness. The guidance recommends that a functional assessment to determine residual physical and non-physical deficits should be performed on patients who have been hospitalized with critical illness before the patient is discharged from hospital. It also recommends that further reviews should be instituted in the community two-to-three months post discharge [23]. This assessment should be performed by clinicians skilled in both critical care and the rehabilitation of the residual deficits.

It is not uncommon for patients to develop a plethora of deficits as a consequence of the deconditioning that often accompany a stay in the ICU. With the COVID-19 situation, current efforts to limit the spread of the viral infection have necessitated the restriction of movement in the community. This movement restriction has the unintended consequence of preventing access to traditional physical face-to-face PR classes. Alternative means and methods to deliver PR and physical reconditioning programs are required. Additionally, these patients do develop significant psychological deficits that also require therapy input and support. Zhao et al. [22] made the following recommendations for PR in COVID-19 patients. 1: For inpatients with COVID-19, respiratory rehabilitation should be directed to relieve the symptoms of dyspnoea, anxiety, and depression and eventually improve physical functions, as well as the quality of life; 2: For severe/critical inpatients, early respiratory rehabilitation is not suggested; 3: For patients in isolation or in the community, respiratory rehabilitation guidance should be conducted through educational videos, instruction manuals, or remote consultation (tele-rehabilitation); 4: Assessment and monitoring should be performed throughout the respiratory rehabilitation process; and 5. Proper grade personal protection equipment (PPE) should be used in implementing any face-to-face contact. 

Traditional (physical face-to-face) PR is beneficial to patients with chronic respiratory disease largely through the improvement in symptoms and functional capacity. This is attributed to improved muscle function rather than direct effects on lung function. It focuses on fitness and strength exercise, breathing exercises, postural drainage positions, patient education, and relaxation training. Pulmonary rehabilitation has been demonstrated to minimize the symptoms of patients with chronic obstructive airway disease (COPD). It can decrease the impact of disability and improve the quality of life [24]. Additionally, it promotes independence and can increase participation in physical and social activities. It also helps to reduce overall health care costs through a reduction in hospitalizations. However, there is an acknowledgement that patients respond differently in terms of benefits [25]. This variability suggests that a one-size-fits-all is unlikely to work for all patients. A bespoke assessment that informs individualized rehabilitation prescription offers the best management plan. This can be augmented with appropriate psychological therapy to address any associated mood deficits. This bespoke rehabilitation approach will be required in managing survivors of COVID-19 who have residual deficits.

## 3. Making a Case for Tele-Rehabilitation and Why It Should Be Considered for COVID-19 Patients

Assessing rehabilitation needs can easily and readily be done remotely through various assessment tools; however, the on-going restriction in terms of movement and the reduced capacity of community rehabilitation services due to limited manpower to actually deliver the required therapy in the community have the effect that the rehabilitation needs, even when identified, might not be fulfilled. Alternative ways of working will have to be explored if the rehabilitation needs of these patients are to be met with a reduced workforce. Tele-rehabilitation programs using appropriate technology have the potential to help deliver therapy in situations such as this. It has been demonstrated to be effective in PR of patients with COPD [26]. It can also be utilized in providing remote support for psychological issues. Tele-rehabilitation use has been most widespread in the field of stroke rehabilitation, where evidence has shown that people who received tele-rehabilitation have similar outcomes for activities of daily living function compared to those who receive face-to-face therapy and usual care [27]. In the current pandemic-related circumstances with their attendant impact on available workforce, tele-rehabilitation offers a quick and readily available means of therapy provision to patients. It can be used to augment therapy delivery to suitable community-based patients.

Based on the documented evidence from previous outbreaks of similar respiratory virus epidemics, many of the discharged patients treated for COVID-19 are likely to have residual deficits affecting physical, psychological, and respiratory function. The evaluation and assessment of these needs will be required to appropriately plan for intervention. In view of the novel nature of this disease, it is anticipated that the clinical expertise to evaluate, assess, and monitor all discharged COVID-19 patients within this group for residual deficits will be limited within community therapy services. Furthermore, the resources to deliver the required intervention within the community will be limited. Though hospital-based services might have the clinical expertise to deliver the required input, the perennial issue of resource limitation makes it impractical for them to deliver the input. However, with tele-rehabilitation, discharged patients in the community can still access this expertise from hospital-based services. There are experienced therapy staff deployed from hands-on patient facing roles (either from personal circumstances such as underlying health conditions or organizational restructuring) during the peak of the pandemic. This group of staff can provide a readily accessible pool of experienced therapists to provide remote assessment and therapy. Tele-rehabilitation offers a means to deliver remotely supervised therapy by experienced clinicians to patients within their homes or community dwellings. 

Tele-rehabilitation is the application of telecommunication technology for supporting rehabilitation services [28], i.e., the delivery of rehabilitation services via information and communication technologies. Clinically, this term encompasses a range of rehabilitation services that include assessment, monitoring, prevention, intervention, supervision, education, consultation, and counselling. The rehabilitation process is a continuous interactive process that requires the frequent monitoring of the patient’s functional ability, which is used to guide and adjust therapy delivery based on the patient’s progress. With the advent of virtual reality, video gaming, video conferencing, and tele-medicine technologies, the concept of tele-rehabilitation has gradually become established and has been demonstrated to be equally as effective compared to traditional face-to-face hands-on therapy delivery model in stroke survivors [29] and also in cardiac patients [30,31], with no adverse effects reported.

The use of remote rehabilitation methods is not new. Countries with isolated communities have had to utilize technology in order to offer equitable access to services and to provide more efficient use of staff time and reduce costs [32]. In the UK, home-based PR [33] and online PR sessions [34] with telephone follow up have not been as effective as face-to-face PR in altering lung function but have demonstrated positive effects on anxiety and the quality of life [35]. This might be due to the monitoring process, as other studies employing video conferencing platforms to enable the therapist to provide remote monitoring and encouragement have proved to be more effective [36] and safe [37]. Some programs also provide exercise equipment such as cycle ergometers to the participants to increase compliance [38]. Such programs have been shown to benefit patients with chronic lung conditions, and though there is currently no published evidence of effectiveness of such programs in COVID-19, it is anticipated that it will be beneficial. 

Kairy et al. [39], in a systematic review of 28 papers on tele-rehabilitation in community-based patients with various conditions (neurological, cardiac, spinal cord injuries, and those with speech-language impairments), observed improved outcomes that are at least similar to or better than alternative interventions. Attendance and compliance were high, and patient satisfaction with tele-rehabilitation was consistently high, though consultation time was observed to be longer. Preliminary evidence of potential cost savings was also noted with tele-rehabilitation interventions. The review concluded on the need for more studies on the efficacy and effectiveness of tele-rehabilitation to help guide resource allocation and policy decision-making.

This emerging field of rehabilitation has the advantage that the therapy can be delivered wherever is most convenient for the patient through the use of modern digital communication. The clinician is able to prescribe an array of rehabilitation programs and monitor adherence in the performance of the tasks. Feedback information, as well as an assessment of how well the patient is performing, can be provided—both in real time and following a review of data. Portability and versatility are two key advantages of tele-rehabilitation over the traditional modes of community therapy delivery. Tele-rehabilitation programs make use of natural body movements with minimal or no constraints from the use of peripheral equipment. This has the advantages that activities can take place in virtually any setting and the exercise program can be designed around the home environment. The use of visualization screens to provide instructions and as means of providing visual feedback makes it engaging and stimulating. Another advantage is that patients can participate in a tele-rehabilitation program regardless of discharge destination. Patients discharged to residential care facilities can also participate as long as they can be supported to follow the programs.

The benefits to healthcare staff include a reduction in therapy space accommodation pressures because tele-rehabilitation only requires a small workspace footprint. Two therapists, one to monitor the screen to ensure patient safety and the other to demonstrate the exercises, could effectively conduct remotely supervised group therapy sessions. Overall, it requires fewer staff than in face-to-face groups, and the attendant reduction in footfalls within the therapy center further helps with maintaining social distancing rules within the workplace. Virtual waiting rooms can be created and monitored remotely by an administrator who has control over a number of clinics, and it is therefore more cost effective.

There are potential drawbacks to tele-rehabilitation. Many of the tele-rehabilitation products on offer have little or no evidence of proven clinical efficacy or effectiveness. Their safety profile rather than clinical efficacy seems to be the main factor guiding their commercial availability. This can potentially restrict patient participation due to lack of access to the chosen technology or platform. Novel digital products and apps can be bewildering for some patients, though using a basic telephone facility as a minimum tool for the screening assessment makes the implementation of remote support and assessment feasible for most services. It is generally assumed nowadays that most people have access to a telephone, though internet access is not universal. A report published by the UK Office of National Statistics in 2019 suggested that only 7% of households across Great Britain do not have internet access. The proportion for the 65 years and older age group is higher, with 24% of them not using the internet in the last three months prior to the survey [40]. A telephone-based screening assessment is therefore likely to provide almost universal coverage in identifying patients that would be suitable to enroll in the other components of the tele-rehabilitation program.

Tele-rehabilitation offers a means to deliver the required therapy input while also maintaining the need for social distancing, which has been implemented as part of the raft of measures to slow the spread of the SARS-Cov-2 virus infection in communities. Tele-rehabilitation facilitates the continuation of a rehabilitation program within a patient’s home, which is often the preferred location by most patients for therapy input [41]. Tele-rehabilitation offers an alternative means to provide PR to community-dwelling patients following discharge from a hospital. It helps to avoid the issue of PPE supply in community settings and allows therapists to deliver therapy input from a safe location. This removes the anxiety and psychological issues associated with face-to-face therapy during the pandemic episode. Given the psychological impact of disease entity, components of the tele-rehabilitation program could include sessions designed by psychologists aimed at enhancing the psychological well-being of the participants.

Home tele-rehabilitation systems are cost effective, especially when primarily used to monitor or evaluate patients during corrective therapy. The loss of physical human contact (face-to-face interaction) with the doctor or therapist is a potential drawback, but, as noted previously, the risk of cross-infection with such a highly contagious infective agent like the SARS-Cov-2 virus mitigates against this disadvantage. Potential factors that might prevent individual patients from accessing tele-rehabilitation program include cognitive impairment, severe pulmonary hypertension, unstable cardiovascular disease, uncontrolled seizure disorder, poor balance or vestibular control, and sensory and communication problems. For these patient cohorts, more traditional approaches enable closer risk management, but, for the majority, tele-rehabilitation offers a means to deliver supervised therapy by experienced clinicians to the patients within their home or community dwelling. 

The British Thoracic Society (BTS) guidelines [42] on PR can be readily incorporated into a tele-rehabilitation program. Based on these guidelines, community-dwelling COVID-19 patients could be managed with telehealth programs to deliver the required rehabilitation input in the form of tele-rehabilitation.

## 4. Proposed Post-Discharge Follow-Up and Tele-Rehabilitation Pathway for COVID-19 Patients 

The COVID-19 pandemic has had a massive impact on the UK National Health Service. Routine care pathways were suspended as part of the efforts geared towards ensuring that the healthcare system was not overwhelmed [43,44]. Rehabilitation services across the primary, secondary, and tertiary tiers of healthcare underwent service modification. Inpatient specialist rehabilitation units were relocated in some hospitals and some rehabilitation medicine clinicians were drafted to assist in the frontline acute care effort. Patient discharge from the rehabilitation units was expedited based on being medically fit for discharge. A social services discharge hub with community assessment was created with the view for ongoing therapy needs to be fulfilled within the community. Patients admitted to hospital with COVID-19 followed two clinical pathway trajectories. Those with severe disease requiring ventilatory support were managed in the intensive care units, and a larger cohort was managed on acute care wards till discharge. The pressure to ensure sufficient capacity within the system necessitated that these patients were discharged from the hospital once they were medically fit. A small but significant minority with severe residual physical deficits were referred to specialist inpatient rehabilitation prior to discharge. Majority underwent a fast-track discharge process back to the community once basic functional needs could be supported [44]. 

**Figure 1:**
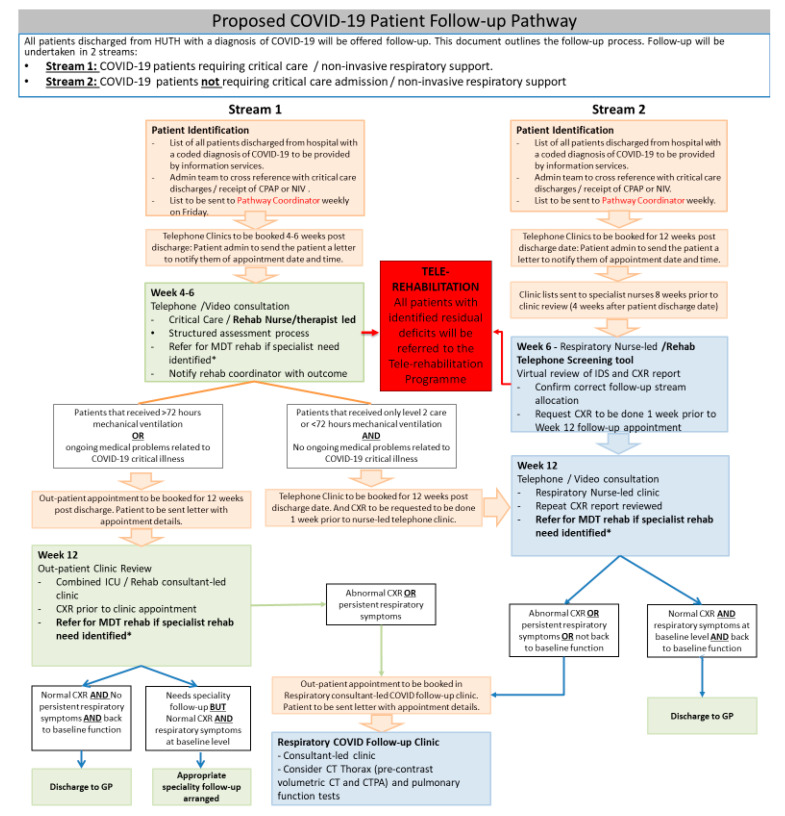
Proposed post-COVID-19 follow-up pathways. CPAP: continuous positive airway pressure; NIV: non-invasive ventilation; MDT: multi-disciplinary team; CXR: chest X-ray; ICU: intensive care unit; CT: computerized tomography; CTPA: computerized tomography pulmonary angiogram; and HUTH: Hull University teaching hospitals. * Specialist rehabilitation needs include but not limited to cognitive impairment, speech and swallowing difficulties, severe mobility and balance issues, and severe mood (anxiety and depression) issues.

**Figure 2:**
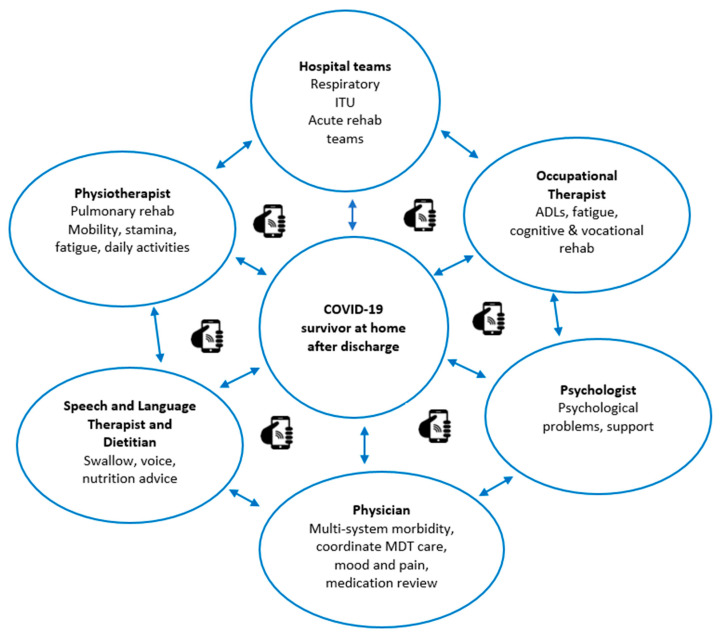
Multidisciplinary tele-rehabilitation model.

In order to ensure that post-discharged COVID-19 patients were appropriately monitored and provided with the required input, a group of clinicians with clinical expertise spanning across intensive care, respiratory medicine, infectious disease, rehabilitation medicine, and therapy disciplines based at the Hull University Teaching Hospital NHS Trust developed the proposed pathway (Figure 1). The initial drive to develop the follow-up process and pathway came from the need to maintain medical monitoring in a completely new disease. Clinical experience, however, indicated that most of the issues affecting the discharged patients were within the realm of rehabilitation medicine. This highlighted the need for all patients to have a rehabilitation assessment. A series of focus group meetings were conveyed using a tele-conferencing platform. A hospital management buy-in into the proposed pathway facilitated the acquisition of vital administrative support to help with patient tracking and scheduling the follow-up appointments. The proposed pathway will provide a multi-level follow-up and assessment process for all patients discharged following inpatient care management for COVID-19. The care pathway aims to evaluate the post recovery rehabilitation and the clinical needs of patients following infection with the SARS-Cov-2 virus. Crucially, the pathway has an embedded multidisciplinary tele-rehabilitation component (Figure 2) to assess and deliver therapy to patients based on the identified needs. An assessment form covering the domains of the international classification of functioning (ICF) has been designed for remote application through the telephone. To ensure coordination and avoid overloading the patients with multiple isolated interventions, a virtual multidisciplinary team (MDT) of various rehabilitation discipline specialists will deliver the core of the tele-rehabilitation program. Each patient identified as requiring MDT input will have access to the team expertise.

Discharged COVID-19 patients will be managed along two streams based on whether they had intensive care input with respiratory support: mechanical ventilation, CPAP (continuous positive airway pressure) or high flow nasal oxygen, or not. Electronic coding will enable patients who had a hospital admission where they tested positive for COVID-19 to be identified to a pathway administrator. The codes also identify which patients require intensive respiratory support (i.e., stream 1) from those who are able to remain on a ward (stream 2). The pathway administrator will receive weekly updates and then book patients into the appropriate assessment clinics. The pathway will incorporate two assessment points at four-to-six weeks and 12 weeks where clinicians make contact remotely with the patients (Figure 1). The four-to-six week assessment will be used to identify suitable patients who may benefit from a tele-rehabilitation program and providing them with the opportunity to enroll. A multidisciplinary rehabilitation telephone screening tool [45] developed by one of the authors (M.S.) will be used for the rehabilitation assessment at the four-to-six weeks post discharge. The telephone screening tool is based on the domains of the ICF [46]. The screening tool was further modified to explore key medical and functional sequelae of COVID-19, as identified in the various guidelines issued by the UK professional bodies for rehabilitation medicine, respiratory medicine, intensive care medicine, and allied healthcare professionals. The screening assessment tool was piloted in both Leeds and Hull as part of a quality improvement program (QIP) to allow for feasibility and a comparison of data trends.

Bespoke interventions tailored to individual circumstances will be provided based on the assessment (Figure 2). The tele-rehabilitation therapy program suite will incorporate the core principles of PR of reducing anxiety relating to breathlessness and additionally optimize the aerobic capacity, strength, endurance, and functional ability of the patients. There will also be an early focus on managing fatigue and pacing, since profound fatigue appears to be a distinct limiting factor in the recovery of these patients. The use of COPD web-based programs and apps was explored in the design of the tele-rehabilitation program. However, as COVID-19 is a novel condition, we wished to retain the ability to respond to identified patient needs in real-time, even if virtually. The pathway was designed to be adaptable, and, as further evidence of clinically effect therapy and treatment of COVID-19 emerges, these programs and apps could be added to the pathway The program will use attend anywhere^®^ [47], an NHS digital-approved secure video conferencing platform to deliver structured exercises supervised by trained therapists to patients identified as requiring such intervention. Supervised exercise sessions will be provided two times each week. Activities will be commenced at mild intensity with progression over subsequent weeks to moderate intensity as tolerated. Other rehabilitation needs identified during the telephone assessment process will trigger referral for specialist interventions. The tele-rehabilitation exercise program will be offered for six-to-eight weeks, and an onward referral to other services will be implemented if required. The implementation of the pathway will be monitored through a service evaluation program, and it is anticipated that formal research studies to explore the feasibility and outcome of the tele-rehabilitation program will be conducted. To ensure the smooth running of the pathway and facilitate the assessments, a coordinator with clinical experience of specialist rehabilitation will be appointed to supervise the pathway and to ensure that the required data are collected for service evaluation.

## 5. Conclusions

Due to the nature of the on-going coronavirus pandemic and its impact on healthcare utilization, it is anticipated that many patients with COVID-19 discharged from hospitals will have residual rehabilitation needs. The evaluation and assessment of these needs will be required to appropriately plan for intervention. It is also anticipated that the resources to deliver the intervention in the community will be limited, thereby necessitating considerations for novel intervention delivery techniques.

Patients should be offered the opportunity to participate in tele-rehabilitation programs in addition to any other input provided by community services. Tele-rehabilitation would be helpful for majority of patients who require input such as PR, psychological support, and nutrition advice. Patients unable to participate in a tele-rehabilitation program and those requiring highly specialist input for impairments such as balance deficits would require face-to-face assessment, and appropriate infection risk management will be implemented to support this

Referrals to services to support with dysphagia, nutrition, balance, cognitive difficulties, psychological support, or other neuromuscular sequelae should still be made as required. Participating in a tele-rehabilitation helps to avoid a gap in service provision following discharge from hospital. Service evaluation should be an integral component of such programs.

We therefore propose that that in exploring the recovery trajectories of COVID-19 patients following discharge from hospital, supervised multidisciplinary tele-rehabilitation programs should be an integral component of the follow-up pathway because they provide an effective modality of assessing and managing residual deficits.
